# Pyrosequencing of 16S rRNA genes in fecal samples reveals high diversity of hindgut microflora in horses and potential links to chronic laminitis

**DOI:** 10.1186/1746-6148-8-231

**Published:** 2012-11-27

**Authors:** Samantha M Steelman, Bhanu P Chowdhary, Scot Dowd, Jan Suchodolski, Jan E Janečka

**Affiliations:** 1 Department of Veterinary Integrative Biosciences, Texas A&M University, College Station, TX, 77843-4458, USA; 2 Molecular Research LP, Shallowater, 79363, TX, USA; 3 Gastrointestinal Laboratory, Texas A&M University, College Station, TX, 77843-4458, USA

## Abstract

**Background:**

The nutrition and health of horses is closely tied to their gastrointestinal microflora. Gut bacteria break down plant structural carbohydrates and produce volatile fatty acids, which are a major source of energy for horses. Bacterial communities are also essential for maintaining gut homeostasis and have been hypothesized to contribute to various diseases including laminitis. We performed pyrosequencing of 16S rRNA bacterial genes isolated from fecal material to characterize hindgut bacterial communities in healthy horses and those with chronic laminitis.

**Results:**

Fecal samples were collected from 10 normal horses and 8 horses with chronic laminitis. Genomic DNA was extracted and the V4-V5 segment of the 16S rRNA gene was PCR amplified and sequenced on the 454 platform generating a mean of 2,425 reads per sample after quality trimming. The bacterial communities were dominated by Firmicutes (69.21% control, 56.72% laminitis) and Verrucomicrobia (18.13% control, 27.63% laminitis), followed by Bacteroidetes, Proteobacteria, and Spirochaetes. We observed more OTUs per individual in the laminitis group than the control group (419.6 and 355.2, respectively, P = 0.019) along with a difference in the abundance of two unassigned Clostridiales genera (P = 0.03 and P = 0.01). The most abundant bacteria were *Streptococcus* spp.*, Clostridium* spp*.,* and *Treponema* spp.; along with unassigned genera from Subdivision 5 of Verrucomicrobia, Ruminococcaceae, and Clostridiaceae, which together constituted ~ 80% of all OTUs. There was a high level of individual variation across all taxonomic ranks.

**Conclusions:**

Our exploration of the equine fecal microflora revealed higher bacterial diversity in horses with chronic laminitis and identification of two Clostridiales genera that differed in abundance from control horses. There was large individual variation in bacterial communities that was not explained in our study. The core hindgut microflora was dominated by *Streptococcus* spp., several cellulytic genera, and a large proportion of uncharacterized OTUs that warrant further investigation regarding their function. Our data provide a foundation for future investigations of hindgut bacterial factors that may influence the development and progression of chronic laminitis.

## Background

The microflora within the gastrointestinal system directly affects energy metabolism, digestive function, mucosal immune system development, and disease pathogenesis of its eukaryotic host [1-4]. This is particularly true for herbivores, including the horse, which are dependent upon fermentation by bacteria to utilize plant structural carbohydrates [4]. Therefore, a detailed knowledge of gut microflora is essential for understanding the nutritional needs of horses and the contribution of gut homeostasis to equine health. Research on bacterial communities has recently flourished with the application of next-generation sequencing (NGS) technology [5]. Studies incorporating NGS have led to the discovery of thousands of novel species (i.e., Operational Taxonomic Units [OTUs]) and elucidation of their ecological function within the gut of vertebrates [5-7]. Numerous factors including the evolutionary history of the host, age, and diet influence the diversity of gut microbes; they in turn have been implicated in a broad range of disorders including Crohn’s disease, chronic diarrhea, inflammatory bowel disease, type I diabetes, obesity, and asthma [2,7-9].

Alterations in hindgut bacterial communities have also been associated with several equine diseases [10-17]. Excess nonstructural carbohydrates (i.e., starches, fructans, or simple sugars) that are not digested in the foregut enter the cecum and colon, where bacterial fermentation produces byproducts including lactic acid and gas, which can cause colic [4,16,17]. The same initiators can also lead to the development of laminitis, which often occurs subsequent to overconsumption of grain or after feeding on lush pasture rich with nonstructural carbohydrates [18-20]. Starch and oligofructose overload-induced models have revealed strong associations between onset of laminitis and proliferation of *Streptococcus* and *Lactobacillus* bacteria, with a concurrent decrease in intraluminal pH [12-15,21-23].

Numerous studies have characterized and enumerated bacteria of the equine hindgut, primarily relying on culturing of bacteria, clone-based sequencing of Polymerase Chain Reaction (PCR) amplicons, denaturing gradient gel electrophoresis (DGGE), fluorescence in situ hybridization (FISH), or gene terminal restriction fragment length polymorphism (T-RFLP) [21,23-32]. The primary microbes detected consisted of Gram-positive bacteria, many of which were associated with the cluster XIVa of Clostridiaceae, *Streptococcus* spp*.*, and *Lactobacillus* spp. [14,22,24,26]. Up to 96% of all observed OTUs could not be assigned, highlighting how little was known about this ecosystem [30].

Recently researchers have begun to apply 454 sequencing of 16S rRNA amplicons to understand the equine gut microflora [10,33,34]. A total of 1,518 OTUs have been observed in feces from just two horses, with Firmicutes, Verrucomicrobia, and Proteobacteria being the most abundant Phyla, and Subdivision 5 *Incertae sedis* spp., TM7 *Incertae sedis* spp., and *Treponema* spp. the most common genera [33]. In a study examining colitis, Firmicutes were found to dominate the feces of normal horses in contrast to Bacteroidetes in horses with undifferentiated colitis [10]. Bacterial communities in the stomach were also found to be dominated by the Phyla Firmicutes, Proteobacteria, and Bacteroidetes, with *Lactobacillus* spp., *Streptococcus* spp., and *Moraxella* spp. comprising the most abundant genera [34]. The stomach microflora segregated based on management (stabled versus pastured) and sampling methods (biopsy versus post mortem) [34]. These studies show a much more diverse assembly of bacteria than previously described; however, the mechanisms linking bacterial diversity to diseases such as colic, colitis, and laminitis are yet to be elucidated.

We thus explored the equine hindgut microflora by pyrosequencing bacterial 16S rRNA gene segments present in feces of normal horses and those suffering from chronic laminitis. Our goals were to (1) describe the level of microbial diversity and (2) compare the microflora of healthy horses to those with chronic laminitis. We hypothesized that horses with chronic laminitis, which had in the past experienced a bout of acute laminitis and presumably a radical shift in bacterial flora that accompanies this disease, would harbor a different microbial population. Our study contributes to the characterization of the equine gut microbiome and its potential link to laminitis.

## Results

### Sequencing depth and alpha diversity

**Figure 1:**
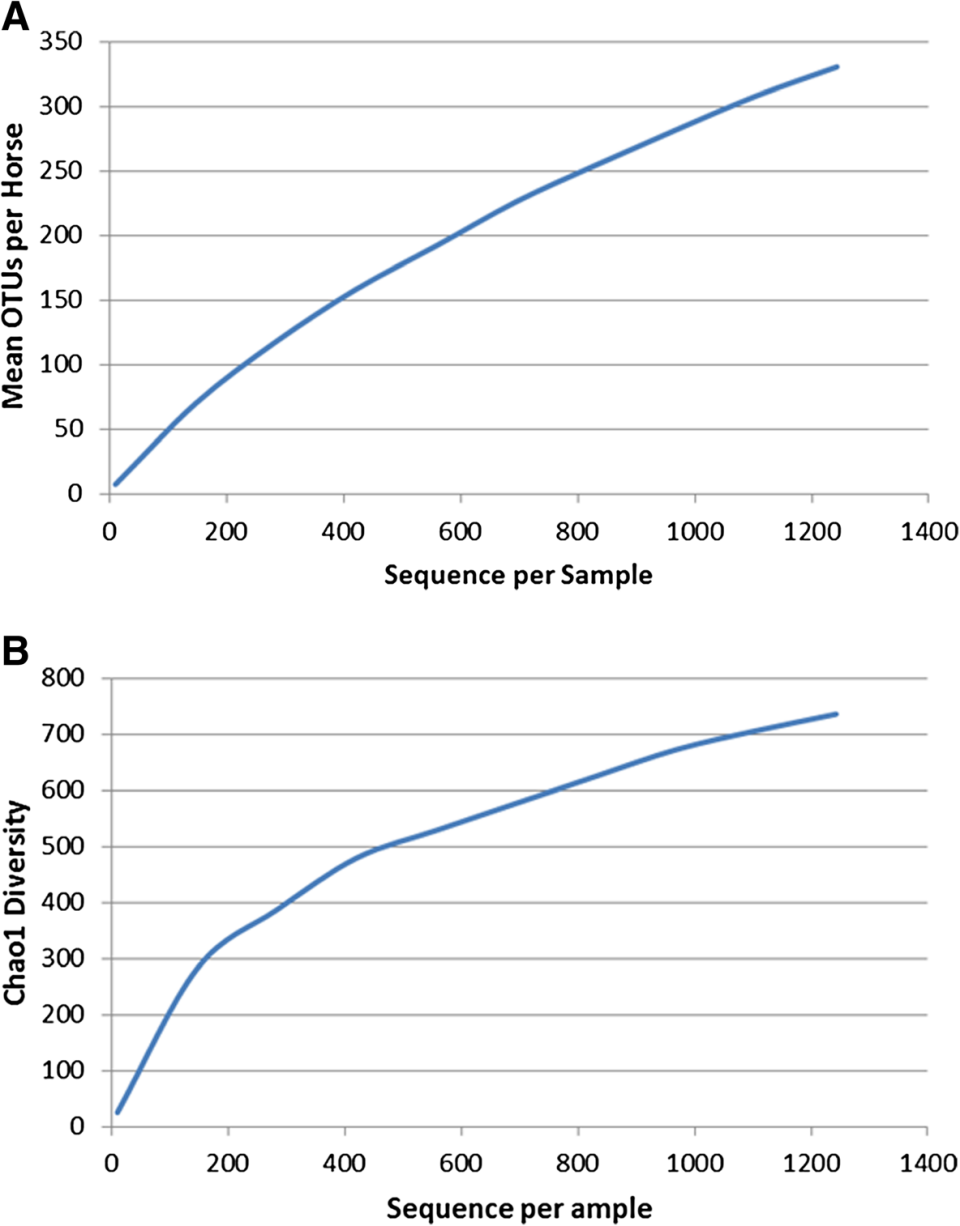
**Observed bacterial OTUs and Chao1 index plots.** Plots were made using data rarefied to a depth of 1,200 reads per sample in QIIME. (**A**) Mean OTUs, (**B**) Chao1 index.

The mean number of reads per sample was 5,159 (range 1,173 – 33,204). When we removed two outliers with the highest depth (12,113 and 30,911 reads) the mean dropped to 2,425 (range 1,032 – 6,578). The 16S rRNA sequences were deposited in the NCBI Sequence Read Archive under the Metagenome BioProject PRJNA177883. One of the horses was a pony and another had recently been on antibiotics so these were not included in the study groups. We separated the horses into 2 groups; those that did not have any history of laminitis (control, n = 9) and those that had chronic laminitis (laminitis, n = 7). We detected a total of 4,894 OTUs in fecal samples from all horses. Of these, 34% (1,660) were identified as chimeras by DECIPHER and excluded from downstream analysis leaving 3,234 OTUs [35]. After the chimeras were removed the mean sequences per sample dropped to 2,204. The laminitis group had a greater number of OTUs per horse than the control group (mean = 419.6 versus 355.2, respectively, P = 0.019) (Table 1). The rarefaction curve of observed OTUs did not plateau with increasing reads suggesting that a higher number of reads per sample would have provided a more comprehensive catalog of bacterial taxa (Figure 1A). However, the Chao1 index of bacterial richness did start to plateau at ~600 reads indicating that the main components of community diversity were detected with our level of depth (Figure 1B). The Chao1 was significantly different between control and laminitis groups (P = 0.020) (Table 1).

**Table 1:** Diversity indices of the gastrointestinal microflora in horses

	**All horses**	**Control**	**Laminitis**
97% Similarity OTUs			
OTUs per animal	381.7 (SE = 26.6)	355.2 (SE = 26.3)	419.6 (SE = 49.3)
OTUs (rfd)*	338.8.0 (SE = 52.3)	330.4 (SE = 57.5)	369.5 (SE = 51.8)
Chao1	795.7 (SE = 51.7)	741.9 (SE = 66.2)	872.6 (SE = 77.4)
Chao1 (rfd)*	761.3 (SE = 82.2)	735.7 (SE = 123.4)	816.4 (SE = 65.5)
Phylogenetic Distance	16.15 (SE = 0.82)	15.37 (SE = 0.85)	17.26 (SE = 1.50)
Phylogenetic Distance (rfd)	14.85 (SE = 1.50)	14.43 (SE = 1.05)	16.00 (SE = 2.00)
Shannon	5.07 (SE = 0.59)	5.01 (SE = 0.71)	5.16 (SE = 1.06)
Simpson	0.74 (SE = 0.08)	0.75 (SE = 0.09)	0.72 (SE = 0.14)
Genera			
Total	108	101	89
Mean per animal	53.4 (SE ± 1.60)	52.3 (SE ± 1.60)	55.0 (SE ± 1.65)

The OTUs (97% similarity), Chao1, Phylogenetic Distance, Shannon, and Simpson diversity indices were estimated in QIIME. Rarified OTU, Chao1, and Phylogenetic Distance estimates were rarefied (rfd) to a depth of 1,100 reads to reduce sampling heterogeneity. * Significant difference, (OTUs, P = 0.019; Chao1, P = 0.020).

### Phylum diversity

**Figure 2:**
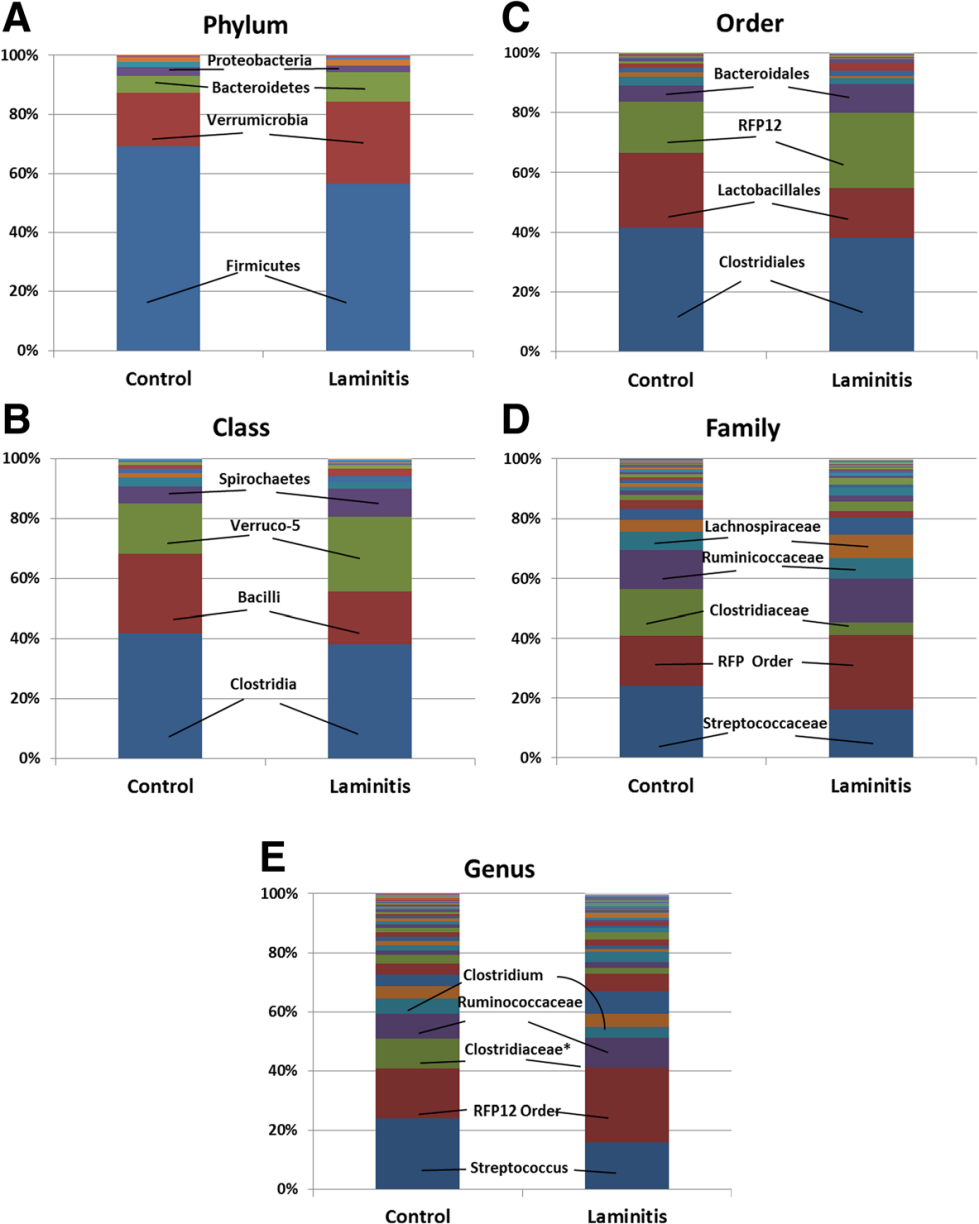
**The abundance of bacterial taxonomic groups in horses.** The mean percentage of reads assigned to the respective taxonomic group for control and laminitis groups. Taxonomic assignments were based on 16S rRNA sequences using the Ribosomal Database Project classifier in QIIME. (**A**) Phylum, (**B**) Class, (**C**) Order, (**D**) Family, (**E**) Genus. *Significant difference between groups (P = 0.03 and 0.01, respectively).

The majority of OTUs belonged to Firmicutes (69.21% control, 56.72% laminitis) (Figure 2A). Verrucomicrobia was next most abundant (18.13% control, 27.63% laminitis) followed by Bacteroidetes (5.71% control, 9.94% laminitis). The remaining 6.95% of the equine bacterial population was either Spirochaetes (2.52%), Proteobacteria (0.95%), or belonged to one of 11 other Phyla (0.13%). Firmicutes was always the most abundant. Verrucomicrobia was the second most abundant in all of the horses, except for the pony and the horse that had received antibiotics within the last 2 weeks. In both these animals, Proteobacteria was the second most abundant Phylum.

### Class diversity

Twenty-nine bacterial Classes were observed in horse feces; only 8 of these contained >1% of all OTUs. Clostridia of the Firmicutes Phylum was the most abundant (41.75% control, 38.15% laminitis) (Figure 2B). In the controls, the second most numerous was Bacilli (26.65%), also in Firmicutes, and third was Subdivision 5 of Verrucomicrobia (Verruco-5) (16.81%); however, this trend was reversed in the laminitis group (17.44% Bacilli and 25.02% Verruco-5). In all but one horse, either Bacilli or Clostridia were the most common. However, large individual variation in abundance was observed; Bacilli varied from 1.11% to 93.57%, Clostridia from 2.22% to 47.42%, and Verruco-5 from 0.86% to 38.19%.

### Order diversity

A total of 44 Orders were detected, however, 82% of all OTUs belonged to only 3 of them; Clostridiales (41.63% control, 37.99% laminitis), Lactobacillales (25.10% control, 16.86% laminitis), and RFP12 of Verruco-5 (16.79% control, 25.02% laminitis) (Figure 2C). Additional Orders with a frequency greater than 1% included Bacteroidales, Spirochaetales, Bacillales, and Verrucomicrobiales. The most common among individuals was either Lactobacillales (in 4 control and 3 laminitis horses) or Clostridiales (in 6 control and 4 laminitis horses). There were large amounts of individual variation in abundance of Orders (e.g., Lactobacillales ranging from 3.18% to 93.53%). The Order Burkholderiales of Proteobacteria was the third most common in the pony (14.64%), yet it was observed in only two other horses at a low frequency (< 0.05%).

### Family diversity

Eighty-two Families were detected among all horses with the most dominant being Streptococcaceae (24.17% control, 16.11% laminitis), followed by an unassigned Family in the RFP12 Order of Verrucomicrobia (16.79% control, 25.02% laminitis), and Ruminococcaceae (13.15% control, 14.40% laminitis) (Figure 2D). Additional abundant Families included Clostridiaceae, Lachnospiraceae, unassigned Bacteroidales, 2 unassigned Clostridiales, Spirochaetaceae, Verrucomicrobiaceae, and Clostridiales Family XIII *Incertae sedi*. Ninety percent of all OTUs where attributed to these 9 Families. There were also large amounts of individual variation at this taxonomic level, with the abundance of the unassigned RFP12 Family ranging from 1.1% to 38.19% and Streptococcaceae from 0.40% to 74.99%. Only 0.80% of all reads were attributed to Lactobacillaceae.

### Genus diversity

A total of 108 genera were identified among the 18 sampled horses, with an average of 53.4 ± 1.60 SE per horse. Nineteen genera were found in > 87% of horses; of these 11 were not assigned to any previously described genus (Table 2). Majority of the genera were observed in only a few of the horses. Eighty-nine were present in less than 20% of the individuals and 58 were detected in only one animal.

**Table 2:** The percentage of OTUs that were assigned to the 20 most abundant microbial genera

**Bacterial genus assignment**	**Percent of all horses with genus present**	**Pooled**	**Control group**		**Laminitis group**	
		**Mean**	**Mean**	**S.E.**	**Mean**	**S.E.**
		**N = 13**	**N = 7**		**N = 5**	
*Streptococcus*	100%	21.00%	24.10%	9.03%	16.03%	8.71%
RFP12 (O)	100%	19.96%	16.79%	5.32%	25.02%	4.01%
Ruminococcaceae (F)	100%	8.95%	8.30%	1.92%	9.99%	1.54%
Clostridiaceae (F)	100%	6.20%	10.03%*	0.50%	0.07%	1.29%
Bacteroidales (O)	100%	5.41%	3.96%	0.64%	7.73%	0.67%
*Clostridium*	100%	4.75%	5.30%	2.87%	3.88%	0.80%
Clostridiales (O)*	100%	4.58%	3.80%	1.03%	5.82%	0.92%
Lachnospiraceae (F)	100%	4.14%	4.07%	1.53%	4.25%	0.90%
*Treponema*	100%	2.58%	2.87%	0.34%	2.10%	0.34%
Clostridiales (O)*	100%	2.27%	1.63%	0.34%	3.29%	0.39%
Ruminococcaceae (F)	100%	1.81%	1.66%	0.60%	2.04%	0.26%
*Akkermansia*	94%	1.80%	1.30%	0.32%	2.61%	0.56%
*Oscillospira*	100%	1.44%	1.51%	0.48%	1.34%	0.13%
*Ruminococcus*	88%%	1.30%	1.53%	0.49%	0.95%	0.22%
Lachnospiraceae (F)	100%	1.16%	1.02%	0.27%	1.39%	0.15%
Clostridiales XIII I. sedis (F)	100%	0.99%	0.50%	0.17%	1.78%	0.13%
Firmicutes (P)	94%	0.93%	0.81%	0.71%	1.12%	0.30%
*Lactobacillus*	88%	0.82%	0.88%	0.05%	0.72%	0.18%
*Staphylococcus*	67%	0.78%	1.27%	0.57%	0.00%	0.00%
*Coprococcus*	88%	0.65%	0.60%	0.27%	0.73%	0.21%

The OTU assignments were made using the Ribosomal Database Project classifier in QIIME. The mean and standard error (S.E.) of the percentage of reads that map to the respective genus is provided. If the OTU was not assigned to a known genus its nearest available taxonomic rank is provided. F = Family, O = Order, P = Phylum. *Significant differences between groups (P = 0.03 and 0.01, respectively).

The dominant genera were *Streptococcus* (21.00% control, 16.03% laminitis), an unassigned genus in the RFP12 Order of Verruco-5 (16.79% control, 25.02% laminitis), and an unassigned genus in the Ruminococcaceae family (8.30% control, 9.99% laminitis) (Table 2, Figure 2E). *Streptococcus* was the most abundant genus in 6 control horses and 3 laminitic horses, while the RFP12 genus dominated most of the other horses. Differences in abundance for the top three genera between control and laminitis groups were not significant (P > 0.21). Twelve of the 20 most abundant genera were unassigned. Among the classified dominant genera were *Strepococcus*, *Clostridium*, *Treponema*, *Akkermansia*, *Oscillospira*, *Ruminococcus*, *Lactobacillus*, *Staphylococcus*, and *Coprococcus*. There were significantly more OTUs attributed to two unassigned Clostridiales genera in the laminitis group compared to the control (P = 0.03 and P = 0.01) (Table 2). Similar to all other levels of classification, there was large individual variation in the abundance of the dominant genera; for example, *Streptococcus* varied from 0.40% to 91.96%, the RFP12 genus from 2.78% to 32.60%, and the Ruminococcaceae genus from 0.36% to 15.72%.

### Species diversity

The short 16S sequences (< 500 bp) generated during this study did not permit reliable species-level assignments. Nonetheless, we examined OTUs with greater than 1% abundance to determine which described species they are most closely related to. We detected the following taxa: *Streptococcus equinus* serotype 3, *Rhodococcus wratislaviensis* oucz59*, Prevotella ruminicola, Clostridium sardiniense, Williamsia muralis, Clostridium chartatabidum, Clostridium orbiscindens*. *Clostridium* had the highest number of species relative to other genera (33).

### UniFrac analysis

**Figure 3:**
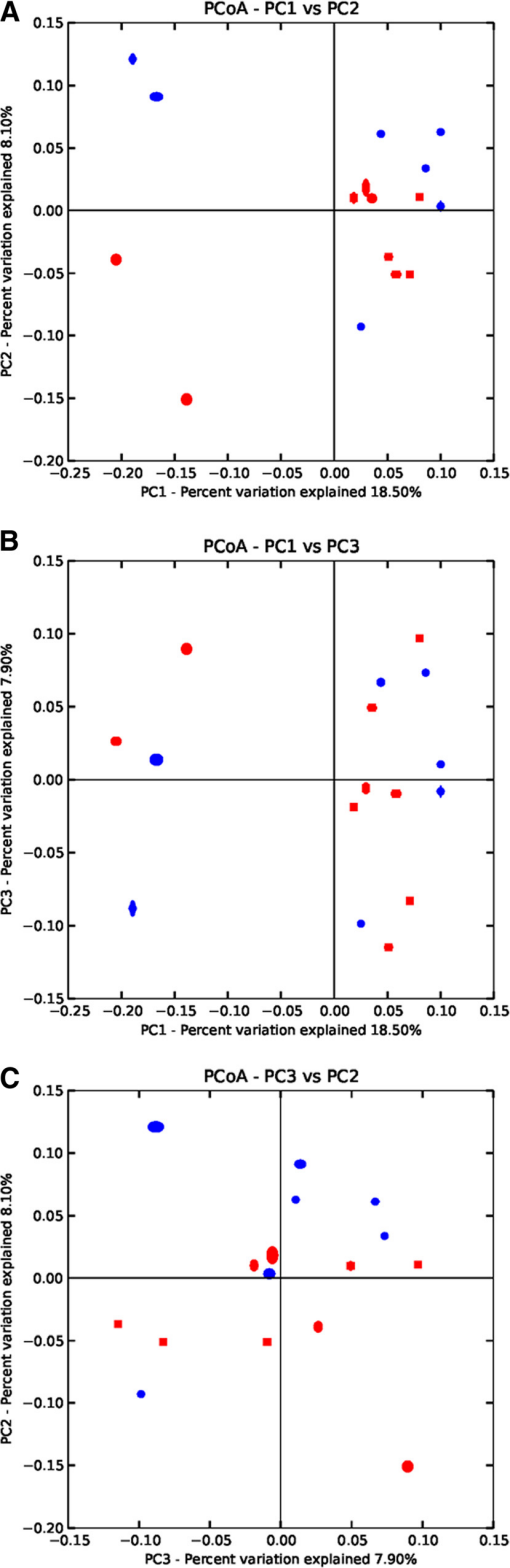
**Principal coordinate analysis of unweighted UniFrac distances.** Principal coordinate analysis (PCoA) plots were made using jackknifed UniFrac distances in QIIME. Red data points represent control horses and the blue horses with chronic laminitis. (**A**) PC1 versus PC2, (**B**) PC1 versus PC3, (**C**) PC2 versus PC3.

Statistical tests dependent on taxonomic categories often fail to detect community level differences in diversity [5]. Approaches that are independent of OTU assignments have thus been developed for comparing microbiomes [36]. We tested the control and laminitis groups for community shifts in the microflora using UniFrac distance, which compares the phylogenetic diversity within groups and is independent of taxonomic classification [37]. To visualize the differences between groups we conducted Principal Coordinate Analysis (PCoA) of weighted and unweighted UniFrac distances and plotted the 3 factors that explained the greatest portion of variation. Jacknifed weighted and unweighted UniFrac distances did not show any significant differences between the two groups (Figure 3A,B,C).

## Discussion

We observed more unique OTUs (3,234) than detected by Shepherd et al. [33] (1,510 OTUs) despite our lower read depth (2,204 versus 28,458, respectively). This is likely because we had a greater number of horses (16 versus 2). However, our Chao1 index of bacterial richness (795.7) and Shannon Index of bacterial diversity (5.07) were lower than in the previous study (2,359 and 6.7, respectively) [33]. There was significantly higher bacterial diversity as estimated from OTUs and the Chao1 index in the laminitis group compared to the control (P = 0.019, P = 0.020, respectively). The only other significant differences between the control and laminitis groups was the higher abundance of two undescribed genera of Clostridiales in the laminitis horses (P = 0.03 and P = 0.01, respectively). This suggests potential changes in bacterial communities that should be further explored.

Our lower bacterial richness and diversity relative to what was previously reported could be attributed to an insufficient number of reads to capture all of the diversity within each sample, particularly for the low abundance OTUs [33]. This is supported by our OTU rarefaction plot that fails to plateau (Figure 1A). Future studies need to generate closer to the 5,000 reads per sample previously recommended [10]. We targeted this level of depth; however, because one of our samples was over-represented (30,911) in the pooled multiplex of amplicons, it reduced the number of reads that were generated for the other samples. Therefore, greater attention needs to be given to DNA extraction, PCR amplification, and library construction so that each amplicon is equally represented.

We successfully assigned a greater number of reads to Phyla (98.42% versus < 62%) than several previous studies using 16S rRNA sequences [24,33]. This is likely because they did not identify and exclude chimeras, which are known to inflate the number of unclassified OTUs [35]. We detected the same number of Phyla (16) as in Shepherd et al.[33], including 4 that were not previously observed in horses; MVP-15, Synergistetes, Chlamydiae, and Deferribacteres [10,33]. The most abundant Phylum we observed in horses, Firmicutes, was also the major component of equine intestinal flora in previous studies that analyzed feces from two adult Arabian geldings [33] and 6 healthy horses [10], stomach contents from 9 hay-fed stabled horses [34], and more traditional studies that used clone-based Sanger sequencing [24,31]. Firmicutes are also common in the gut of other diverse taxa, from cats, dogs, and polar bears to cattle [38-40]. In contrast, Bacteroidetes was the most abundant Phylum among horses that had colitis, supporting the hypothesis that Firmicutes play an important role in gut function [10].

Verrucomicrobia, Bacteroidetes, and Proteobacteria represented the next largest components of the equine gut microbiome that we observed; a pattern similar to previous studies, although the Phyla were not always in the same Order [10,31,33]. We detected higher levels of Verrucomicrobia than previously reported (21.78% versus < 5%) [10,24]. The abundance of this Phylum in horses from central Texas suggests it plays a more important role in hindgut function than previously appreciated. Our second most abundant genus among all horses was an unknown type within the RFP12 Order of Verrucomicrobia. This is a good candidate for culturing in order to classify it and characterize this taxa’s metabolic function.

The cecum and colon of the horse are important for the breakdown of structural carbohydrates and production of volatile fatty acids [4]. Therefore, we expected to detect bacteria known to play such a role, including *Ruminococcus* spp., *Fibrobacter* spp., *Eubacterium* spp., and *Treponema* spp. [24,41]. We indeed detected all of the above; *Ruminococcus* had a mean of 1.03%, *Fibrobacter* 0.042%, *Eubacterium* 0.004%, and *Treponema* 2.18%. Our values were consistent with what has been previously observed (0.50% – 4.4%, 0.01% – 0.75%, 0.09%, 1.90% – 3.00%, respectively) [10,24,33,41]. Interestingly, among the most abundant were unassigned genera of Ruminococcaceae that together composed 8.75% of all OTUs. These may represent important uncharacterized cellulytic bacteria and warrant further investigation.

A vast amount of individual variation was observed in horses at all taxonomic levels. A large portion of this likely came from environmental heterogeneity and differences in animal history, combined with lack of sequencing depth. However, similar individual variation in the equine gut microflora was previously observed. For example, in a study that had a mean of 4,712 reads per sample Bacteroidetes varied from 9.0% to 21.3% and Proteobacteria from 0.0% to 42.7% [10]. Such large individual variation may be a natural trait of equine gut communities; however, the lack of detailed studies using a large number of horse samples limits the inferences that can be made from these patterns.

The genera previously found dominating the lower intestinal microflora in two Arabian geldings based on 16S rRNA pyrosequencing of fecal samples included *Blautia* spp., *Fibrobacter* spp., Subdivision *5 Incertae sedis* spp., TM7 *Incertae sedis* spp., *Treponema* spp., and *Ruminococcus* spp. [33]. In contrast, fecal analysis of a more diverse group of horses found the primary genera *Clostridium* spp., *Coptotermes* spp., *Enterococcus* spp., *Fusobacterium* spp.*, Porphyromonas* spp., *Pseudomonas* spp., and *Prevotella* spp. [10]. A study that used intestinal samples detected many unassigned genera affiliated with *Clostridium* spp., *Butyrivibrio* spp., *Ruminococcus* spp., and *Eubacterium* spp. [24]. We detected all of the above except *Coptotermes* spp., *Porphyromonas* spp., and *Pseudomonas* spp*.*. Among the 14 genera that we observed with > 1.0% abundance were *Streptococcus* spp., *Akkermansia* spp., and *Oscillospira* spp., and 8 genera that could not be assigned to any described genus. This large proportion of unassigned genera among highly abundant OTUs highlights the need for more traditional studies characterizing bacteria and their phenotypic traits to better understand the function of the equine hindgut microflora.

Within abundant genera we found evidence suggesting additional diversity. The most diverse genus was *Clostridium*, which exhibits a wide range of functions and contains both beneficial and pathogenic representatives [42]. For example, *C. botulinum* causes botulism as well as productivity problems and *C. difficile* leads to severe diarrhea and colitis in both humans and livestock [43,44]. Yet, many *Clostridium* spp. are cellulytic and important for the digestion of plant material [45,46]. We detected 33 species of *Clostridium*, including *C. botulinum* in one horse. The population dynamics of bacterial species and their interactions can influence normal gut function and the development of diseases [2]. It is possible that some of the bacterial shifts that affect disease states such as laminitis occur at the species level.

There are numerous lines of evidence suggesting hindgut microflora play a role in the development of laminitis. Several studies have examined the bacterial response during various experimental laminitis models [12-14,21-23]. An estimated 53% of acute laminitis cases occur after overconsumption of grain or grass rich with nonstructural carbohydrates (i.e., starch, fructans, or simple sugars) [20], which is also associated with an explosive proliferation of *Streptococcus* spp. and *Lactobacillus* spp. in the cecum and a concurrent decrease in the intraluminal pH [1,12,15]. Potentially, either of these may be a factor in laminitis. We found remarkable variation in *Streptococcus* spp. among healthy horses (0.40% to 91.96% of all OTUs); therefore the absolute abundance of *Streptococcus* spp. might not be important relative to other changes disrupting hindgut equilibrium.

In the carbohydrate overload model of laminitis, Garner et al. [23] found that *Lactobacillus* spp. increased in abundance by a factor of 10^5^. These changes led to decreased intraluminal pH through the production of lactic acid, which caused death and lysis of other bacterial species including *Enterobacteriaceae* spp. and *Bacilli* spp. [23]. Garner hypothesized that these release endotoxins and cause mucosal damage, contributing to the development of laminitis. Endotoxins can escape into the bloodstream and cause immune system activation, inflammation, fever, low blood pressure, and high respiration rate; some of these symptoms appear during the early stages of laminitis [47-49]. We found *Lactobacillus* spp. represented a small portion of the bacterial communities in the horses we sampled (0.82% controls, 0.60% laminitis). However, we only obtained samples from horses that had a previous history of this condition and not immediately after a relapse of laminitis. Therefore we would not have detected any previous transient *Lactobacillus* spp. proliferation. In addition, we sampled the microflora using feces, an approach which could potentially mask changes occurring in the stomach, cecum, and upper colon [32]. The effects *Lactobacillus* spp. and *Streptococcus* spp. proliferation has on the equine gut microbiome following an increase in dietary nonstructural carbohydrates and relapse of chronic laminitis should be explored.

The composition of the hindgut microflora also has large impacts on feed digestibility and equine nutrition because the horse depends upon microbial fermentation to digest plant structural carbohydrates [4]. Similar to previous studies we found that majority of the abundant bacterial genera were anaerobic fermenters, suggesting that the hindgut microflora are specialized for breaking down plant material. Alterations to bacterial communities may confer advantages to horses under certain dietary conditions [50]. For example, gradual addition of grain into the diet increases the ratio of propionate to acetate, presumably by altering the bacterial microflora [51]. Propionate can be directly converted to glucose and thus this shift is beneficial for horses with high energy needs [4]. However, grain also has more simple sugars, which increase the risk of colic and laminitis [13,17,21]. Future studies should explore how bacterial diversity and function can mediate adaptation to high-energy diets and reduce disease risks.

## Conclusion

Our exploration of the equine hindgut microflora revealed higher levels of bacterial diversity in horses with chronic laminitis and identification of two Clostridiales genera that differed in abundance from control horses. We observed large individual variation suggesting that bacterial populations may be influenced by factors such as genetic background, age, diet, feeding time, and body condition, which were not taken into account during this study. There was high abundance of cellulytic bacteria, primarily Ruminococcaceae and Clostridiaceae. We observed numerous abundant uncharacterized genera within Subdivision 5 of Verrucomicrobia, Clostridiales, and Ruminococcaceae that warrant further investigation into their function. Vast individual differences in *Streptococcus* abundance among healthy horses suggested that this genus is likely not closely linked with chronic laminitis. We recommend studies make efforts to reduce experimental variation by using more homogenous horse populations and incorporating rigorous normalization during 454 library construction to increase the sensitivity for biologically-relevant changes in bacterial communities.

## Methods

### Sample collection

Fresh fecal samples were collected from 10 healthy horses and 7 horses and 1 pony with chronic laminitis. One of the control horses had received antibiotics 2 weeks prior to sampling and therefore we did not include it in our control group. We also excluded the pony from the chronic laminitis group to reduce potential breed-specific differences. Horses were diagnosed as having chronic laminitis by a licensed veterinarian based on clinical presentation, case history, and radiographic evidence of dorsopalmar rotation of the distal phalanx. Horses were kept on two different farms in Brazos County, Texas. All horses had been resident at their respective farms for at least 6 months and had not experienced any recent changes in diet or housing conditions. All animals were maintained on a pelleted concentrate feed containing either 12% or 16% crude protein (Producer’s Co-op, Bryan, TX) in addition to coastal bermudagrass hay and limited amount of alfalfa hay. Detailed information about horses and concentrate feed composition may be found in Additional file 1: Tables S1 and S2. Horses were kept in stalls, large dry lots, or a combination of both. All horses had ad libitum access to water. Only naturally voided fecal samples were collected and therefore did not require an IACUC Animal Use Permit. A single sample was collected from each horse within 3 hours after the morning feeding. As feed takes approximately 28–46 hours to travel through the digestive tracks of horses, the microflora sampled from feces would not be influenced by feeding just prior to collection. Every attempt was made to collect samples immediately after defecation. After collection, samples were stored on wet ice for transport to the laboratory and frozen at −20°C.

### DNA extraction and pyrosequencing

The DNA was extracted from feces using the phenol:chloroform:isoamyl alcohol method after disrupting the starting material with bead beating as described in Suchodolski et al.[52]. The V5-V9 region of 16S rRNA gene was pyrosequenced on the Roche 454 FLX-Titanium instrument (Roche Applied Science, Indianapolis, IN) by the Research and Testing Laboratory (Lubbock, TX) as previously described, with Titanium chemistry modifications [38,53]. Briefly, a 570-bp segment of 16S rRNA was PCR amplified using the HotStarTaq Plus Master Mix Kit (Qiagen, Valencia, CA), 100 ng of template DNA, and universal Eubacterial primers that target majority of GI bacteria: 939 F-TTGACGGGGGCCCGCAC and 1492R-TACCTTGTTACGACTT [54,55]. The exact span of the amplicon in relation to *Streptococcus equinus* strain ATCC 9812 16S rRNA complete sequence is 823 bp to 1409 bp (GenBank Accession NR_042052.1). The thermal conditions were 94°C denaturation for 3 min, 32 cycles of 94°C for 30 sec, 60°C for 40 sec, 72°C for 1 min, and a final 5-min elongation step at 72°C. Subsequently, a second PCR was performed on the above PCR products using the same conditions, but with modified fusing primers that had tag sequences added on the 5’ ends (i.e., LinkerA-Tags-939 F and LinkerB-1492R) to enable multiplexed 454 FLX amplicon pyrosequencing. This secondary PCR was used to incorporate tags and linkers into the 16S rRNA amplicons to avoid unbalanced amplification from the DNA samples. The final amplicons from different samples were mixed in equal volumes, purified using Agencourt AMPure XP beads (Agencourt Bioscience Corporation, Danvers), and sequenced on the 454 platform [38,53].

### Sequence analysis

Species-level operational taxonomic unit (OTU) assignments (>97% similarity, equal to number of matching nucleotides divided by the length of the shorter sequence [56]) were made after trimming positions with < Q25 quality score and discarding reads < 200 bp [56-58]. Sequences were depleted of chimeras and assignments to putative species (>97% similarity) were done with BlastN [58] against a manually curated database compiled from NCBI by the Research and Testing Laboratory (Lubbock, TX) [38]. However, because species-level bacterial assignments using short, single-gene segments are not robust we only used this information to obtain an overview of the potential species present. The main comparisons of microbial diversity within and among horses were made for genus and higher-level classifications as described below.

The statistical analysis of alpha and beta diversity was done from taxonomic classifications and phylogenetic-based methods (UniFrac) not dependent on OTU assignments [59]. The QIIME pipeline with standard scripts and default settings was used for taxa assignments (genus and higher), diversity estimates (OTUs, Chao1 index, phylogenetic distance index, Shannon index, and Simpson index), and phylogeny-based analyses using UniFrac [37,59,60]. Barcodes were removed and the reads trimmed of bases with quality score below Q25; reads with length < 200 bp or any ambiguous bases were removed from dataset. The remaining sequences were clustered using UCLUST with the *furthest* algorithm based on >97% similarity to define OTUs [56]. Representative sequences were selected with the most abundant criteria. Chimeras were identified among the OTUs using DECIPHER and excluded from all subsequent analysis [35]. Taxonomic assignments of the OTUs were made down to the genus level (>95% similarity) using the Ribosomal Database Project (RDP) classifier and the Greengenes reference core set “gg97_otus_4feb2011_aligned.fasta” available from http://greengenes.lbl.gov/cgi-bin/nph-index.cgi[61,62]. Sequences were added to this reference alignment [62] with PyNAST and the alignment was optimized, then filtered to exclude sites with only gaps and excessively variable sites [62,63]. A neighbor-joining phylogeny was reconstructed using FastTree for UniFrac analysis of beta diversity [64].

Rarefied OTU tables were generated to reduce sampling heterogeneity for observed OTUs and Chao1 index and tested for significant differences in QIIME. Unpaired t-tests were used to compare abundance of taxonomic groups between control and laminitis groups. Beta diversity was compared between control and laminitis groups using weighted and unweighted UniFrac phylogenetic-based distances. Principal Coordinate Analysis (PCoA) transformed the UniFrac distances into coordinates that explain the greatest amount of variation. The differences were visualized with 2D and 3D PCoA plots. To minimize sampling bias we rarified OTU matrices using the smallest number of reads observed in any one horse before conducting the UniFrac and PCoA analyses.

## Competing interests

The authors declare that they have no competing interests.

## Authors’ contributions

SMS collected samples, performed some of the data analysis, and drafted manuscript. BPC assisted in experimental design, data interpretation, and manuscript preparation. SD performed PCR amplification, pyrosequencing, and data analysis. JS performed DNA extractions and contributed to development of project. JEJ developed project, performed data analysis, and drafted manuscript. All authors read and approved the final manuscript.

## Supplementary Material

Additional file 1 **Table S1.** Description of horses in each study group. The exact age of all horses in the laminitis group was not known; one horse was estimated to be 7 years of age, the others were over 15 years. None of the horses with laminitis exhibited signs of Cushing’s syndrome, although the initial cause of laminitis was not known for all animals. Abbreviations are as follows: BCS – body condition score [65], QH – Quarter horse, WB – Warmblood, TB – Thoroughbred. Table S2 Feed analysis. Comparison of guaranteed feed analysis of 12% concentrate pellets and 16% concentrate pellets used in this study. Pellets were formulated by Producer’s Co-op (Bryan, TX) and further information is available online: http://www.producerscooperative.com/productsservices/feednutrition/feeds/horse. Click here for file
