# Supplementary material for: Pyrosequencing of 16S rRNA genes in fecal samples reveals high diversity of hindgut microflora in horses and potential links to chronic laminitis
Source: BMC Vet Res. 2012 Nov 27;8:231. doi: 10.1186/1746-6148-8-231 (PMC3538718; doi:10.1186/1746-6148-8-231)
Supplement: Additional file 1 — Table S1. Description of horses in each study group. The exact age of all horses in the laminitis group was not known; one horse was estimated to be 7 years of age, the others were over 15 years. None of the horses with laminitis exhibited signs of Cushing’s syndrome, although the initial cause of laminitis was not known for all animals. Abbreviations are as follows: BCS – body condition score [65], QH – Quarter horse, WB – Warmblood, TB – Thoroughbred. Table S2 Feed analysis. Comparison of guaranteed feed analysis of 12% concentrate pellets and 16% concentrate pellets used in this study. Pellets were formulated by Producer’s Co-op (Bryan, TX) and further information is available online: http://www.producerscooperative.com/productsservices/feednutrition/feeds/horse. [file 1746-6148-8-231-S1.doc]

**Additional files**

**Supplementary Table 1 Description of horses in each study group.** The exact age of all horses in the laminitis group was not known; one horse was estimated to be 7 years of age, the others were over 15 years. None of the horses with laminitis exhibited signs of Cushing’s syndrome, although the initial cause of laminitis was not known for all animals. Abbreviations are as follows: BCS – body condition score , QH – Quarter horse, WB – Warmblood, TB – Thoroughbred.

| **Category** | | **Control** | **Laminitis** |
| --- | --- | --- | --- |
| Number | | 10 | 8 |
| Age (mean) | | 7.7 | >15 |
| BCS (mean) | | 5.0 | 5.0 |
| Breed | |  |  |
|  | QH | 10 | 3 |
|  | Arabian | 0 | 2 |
|  | WB | 0 | 1 |
|  | pony | 0 | 1 |
|  | TB | 0 | 1 |
| Farm | |  |  |
|  | A | 9 | 1 |
|  | B | 1 | 7 |
| Diet | |  |  |
|  | 12% pellet | 0 | 8 |
|  | 16% pellet | 10 | 0 |
|  | coastal hay | 10 | 8 |
|  | alfalfa | 6 | 0 |

**Supplementary Table 2 – Feed analysis**

Comparison of guaranteed feed analysis of 12% concentrate pellets and 16% concentrate pellets used in this study. Pellets were formulated by Producer’s Co-op (Bryan, TX) and further information is available online: <http://www.producerscooperative.com/productsservices/feednutrition/feeds/horse>

| **Component** | **12% Pellet** | **16% Pellet** |
| --- | --- | --- |
| Crude Protein, min | 12.00% | 16.00% |
| Crude Fat, min | 6.00% | 6.00% |
| Crude Fiber, max | 10.00% | 9.00% |
| Calcium, min | 0.65% | 0.60% |
| Calcium, max | 0.85% | 1.00% |
| Phosphorous, min | 0.50% | 0.50% |
| Copper, min | 45 ppm | 45 ppm |
| Selenium, min | 0.30 ppm | 0.30 ppm |
| Zinc, min | 117 ppm | 110 ppm |
| Vitamin A, min | 2,500 IU/LB | 2,500 IU/LB |
